# The effect of music therapy on the anxiety, depression and sleep quality in intensive care unit patients: A protocol for systematic review and meta-analysis

**DOI:** 10.1097/MD.0000000000028846

**Published:** 2022-02-25

**Authors:** Dan Li, Yunhua Yao, Jia Chen, Gang Xiong

**Affiliations:** a The Central Hospital of Enshi Tujia and Miao Autonomous Prefecture, Enshi, Hubei Province, China; b Emergency Intensive Care Unit, The Central Hospital of Enshi Tujia and Miao Autonomous Prefecture, Enshi, Hubei Province, China.

**Keywords:** anxiety, depression, intensive care unit, meta-analysis, music therapy, protocol, sleep

## Abstract

**Background:**

Music therapy serves as a non-pharmacological intervention for a variety of disorders with promising results. However, the effect of music therapy on improving anxiety, depression, and sleep quality in intensive care unit (ICU) patients remains unclear. This meta-analysis aims to evaluate the effect of music therapy on improving anxiety, depression, and sleep quality in ICU patients, thus providing evidences to support music therapy as a novel complementary alternative therapy.

**Methods:**

Randomized controlled trials (RCTs) reporting the efficacy of music therapy on improving anxiety, depression and sleep quality in ICU patients published before January 2022 will be searched in online databases, including the PubMed, the Cochrane Library, Web of Science, Embase, Chinese Biomedical Literature Database, China National Knowledge Infrastructure, VIP Database, and WanFang Database. Literature screening, data extraction, and evaluation of risk of bias will be independently performed by two investigators. Meta-analysis will be performed using Stata 14.0 software.

**Results:**

The results of this meta-analysis will be submitted to a peer-reviewed journal for publication.

**Conclusion:**

This study will provide reliable evidence-based evidence for the effect of music therapy on anxiety, depression, and sleep quality in ICU patients.

**Ethics and dissemination:**

Ethical approval was not required for this study. The systematic review will be published in a peer-reviewed journal, presented at conferences, and shared on social media platforms.

**OSF Registration number:**

DOI 10.17605/OSF.IO/EXAZ6.

## 1 Introduction

Intensive care unit (ICU) is a critical department in a hospital and a key institution for the treatment of critically ill patients.^[1]^ It has unique scenes, including isolation from relatives and friends, influences by patients in adjacent beds, closed environment, stuffy and tense atmosphere, and others, all of which are undesirable stimuli that pose negative psychosomatic effects on critically ill patients or even aggravate their condition.^[2]^ In addition, the conditions of patients admitted to ICU are often serious and complex, which has a great physical and psychological impact on them.^[3]^ It is shown that ICU patients suffer from various disturbances like sleep disorders, anxiety and depression, which not only affect the therapeutic efficacy, but also negatively influence their recovery.^[4,5]^

Good sleep and emotional stability are important for the recovery of ICU patients.^[6]^ At present, intervention measures for anxiety, depression and sleep in ICU patients are mainly divided into pharmacological and non-pharmacological interventions. The latter, such as music therapy, can alleviate anxiety and depression and improve sleep quality. Through mobilizing the memory, imagination and associative ability, music therapy evokes empathy, arises resonance, strengthens positive and positive emotions, eliminates negative emotions, relieves physical stress, and psychological distortion and tension.^[7]^

Several randomized controlled trials (RCTs) have investigated the effect of music therapy on improving critically ill patients.^[8–12]^ Despite the differences in recruitment criteria and the type of music therapy, evidences have all indicated the efficacy of music therapy on improving the anxiety, depression, and sleep quality in critically ill patients. However, the application of music therapy in ICU patients for improving the anxiety, depression and sleep quality lacks evidence-based medical evidence. This study aims to conduct a meta-analysis to synthesize evidences from RCTs reporting the effect of music therapy on improving anxiety, depression, and sleep quality in ICU patients.

## 2 Methods

### 2.1 Protocol

Under the guidance of the preferred reporting items for systematic reviews and meta-analysis protocols (PRISMA-P), this protocol of systematic review and meta-analysis has been drafted.^[13]^ The research framework has been registered on the open science framework (Registration Number: DOI 10.17605/OSF.IO/EXAZ6).

### 2.2 Ethics

Since this is a protocol without patient recruitment and personal information collection, the approval of the ethics committee is not required.

### 2.3 Eligibility criteria

#### 2.3.1 Types of studies

RCTs of music therapy on anxiety, depression, and sleep quality in ICU patients will be included.

#### 2.3.2 Types of participants

1. Older than 18 years;
2. Admitted to the ICU ≥48 hours;
3. Glasgow Coma Scale score ≥14 points.

#### 2.3.3 Types of interventions

Patients in the control group will be given standard care combined with other interventions or not, and those in the experimental group will receive music therapy combined with standard care.

#### 2.3.4 Types of outcome measurements

1. Anxiety scores will be graded by the Self-Rating Anxiety Scale and Baker Anxiety Scale;
2. Depression scores will be graded by the Beck Depression Inventory and Self-Rating Depression Scale;
3. Sleep quality will be assessed by the Pittsburgh sleep quality index.

### 2.4 Exclusion criteria

1. Studies with incomplete data;
2. Republished literatures;
3. Reviews, case reports, letters to the editor, and editorials.

### 2.5 Searching strategy

RCTs reporting the efficacy of music therapy on improving anxiety, depression and sleep quality in ICU patients published before January 2022 will be searched in online databases, including the PubMed, the Cochrane Library, Web of Science, Embase, Chinese Biomedical Literature Database, China National Knowledge Infrastructure, VIP Database, and WanFang Database. Searching strategy in the PubMed was shown in Table 1.

**Table 1:** PubMed search strategy.

Number	Search terms
#1	Music Therapy[MeSH]
#2	Therapy, Music[Title/Abstract]
#3	Music therapy[Title/Abstract]
#4	or/1–3
#5	Intensive Care Units [MeSH]
#6	Care Unit, Intensive[Title/Abstract]
#7	Care Units, Intensive[Title/Abstract]
#8	Intensive Care Unit[Title/Abstract]
#9	Unit, Intensive Care[Title/Abstract]
#10	Units, Intensive Care[Title/Abstract]
#11	ICU[Title/Abstract]
#12	or/5-10
#13	Randomized Controlled Trial[MeSH]
#14	Clinical Trials, Randomized[Title/Abstract]
#15	Controlled Clinical Trials, Randomized[Title/Abstract]
#16	Trials, Randomized Clinical[Title/Abstract]
#17	Random∗[Title/Abstract]
#18	or/13–17
#19	#4 and #12 and #18

### 2.6 Data screening and extraction

**Figure 1:**
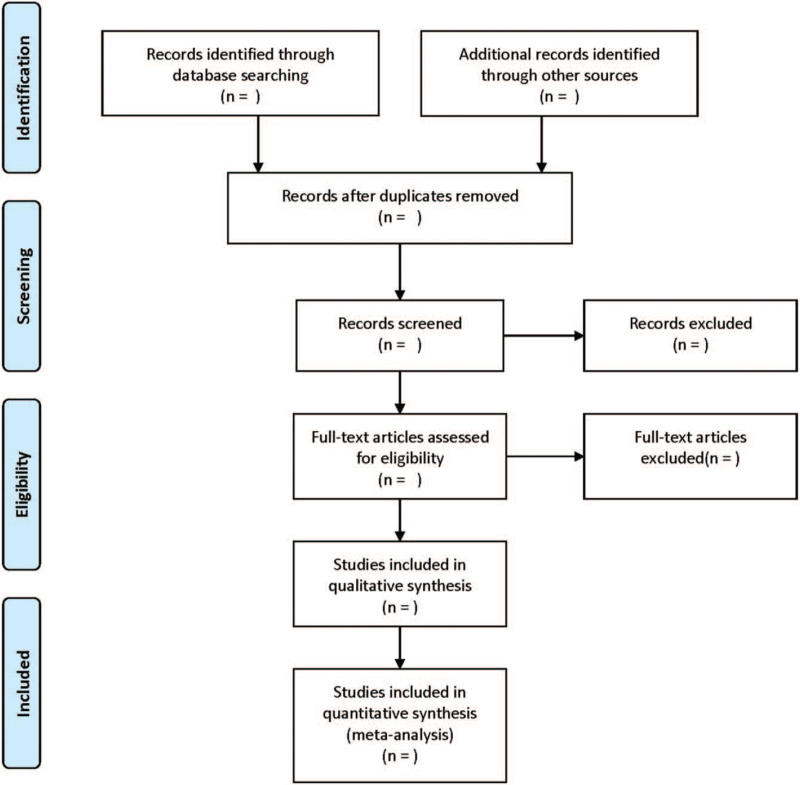
Flow diagram of literature retrieval.

The literature selection process was listed in Figure 1. Literature screening, data extraction and cross check will be independently performed by 2 investigators. Any disagreement will be solved through discussion or consultation with the third investigator. Through reading the title, irrelevant literatures will be initially excluded, and the remaining will be further reviewed by reading the abstract and full-text. If necessary, the original study authors will be contacted by email or telephone to obtain missing data. The following information will be extracted:

1. Baseline information about the included studies: study title, first author, journal of publication, etc;
2. Baseline characteristics of the study population and interventions;
3. Key elements of risk of bias assessment; and
4. outcome measures.

### 2.7 Quality evaluation

The risk of bias in the included studies will be independently evaluated by 2 investigators, and the risk of bias in the cross-checked results will be evaluated using the RCT risk assessment tool recommended by Cochrane handbook 5.1.0.^[14]^

### 2.8 Statistical analysis

Stata 14.0 software will be applied for the meta-analysis. The pooled effects will be estimated using the standardized mean differences (SMDs) and the corresponding 95% confidence interval (95%CI). Heterogeneity between studies will be assessed by I-square (*I*^2^) and *Q*-statistic (*P* < .10), and *I*^2^ > 50% will be recognized as heterogeneity.^[15]^ If *P *≥ .1 and *I*^2^ ≤ 50%, a fixed-effect model will be used for analysis: Otherwise, a random-effect model will be used.

#### 2.8.1 Dealing with missing data

Missing data will be obtained by contacting the author through phone or email before analysis. If the author cannot be reached, or the author has lost relevant data, the meta-analysis will not be conducted.

#### 2.8.2 Subgroup analysis

Subgroup analysis based on timing of intervention, type of intervention, and severity of disease will be performed.

#### 2.8.3 Sensitivity analysis

Sensitivity analysis will be performed by eliminating one study at each time and analyzing the remaining.

#### 2.8.4 Publication bias

If the number of included studies is no <10, Egger regression test and funnel plot will be used to evaluate potential publication bias.^[16,17]^

## 3 Discussion

Music therapy is an emerging therapeutic tool that is acceptable to most patients. It is an economical and safe nursing treatment that has attracted widespread attention from healthcare professionals at home and abroad.^[18–21]^ Patients in ICU are often in serious and complex conditions, thus causing great physical and psychological impact on them.^[22]^ Music therapy for critically ill patients can effectively relieve negative emotions, improve their loneliness, anxiety and depression, and promote rapid recovery from illness. However, whether music therapy can improve anxiety, depression and sleep quality in ICU patients lacks a consensus. As a low-cost and easily accepted intervention method in the field of psychological reconstruction of ICU patients, this meta-analysis aims to analyze the effectiveness of music therapy on improving mental health and sleep quality in ICU patients, which will obtain conclusions about the effects of music therapy on sleep, anxiety and depression in ICU patients.

## Author contributions

**Conceptualization:** Gang Xiong, Dan Li.

**Data curation:** Dan Li.

**Formal analysis:** Dan Li.

**Funding acquisition:** Gang Xiong.

**Methodology:** Yunhua Yao.

**Project administration:** Gang Xiong.

**Software:** Yunhua Yao, Jia Chen.

**Supervision:** Gang Xiong.

**Validation:** Yunhua Yao, Jia Chen.

**Visualization:** Yunhua Yao, Jia Chen.

**Writing – original draft:** Gang Xiong, Dan Li.

**Writing – review & editing:** Gang Xiong, Dan Li.
